# F176. CLINICAL CORRELATES OF CORTICAL STRUCTURE IN ANTIPSYCHOTIC-NAïVE SCHIZOPHRENIA PATIENTS BEFORE AND AFTER SIX-WEEK TREATMENT WITH A DOPAMINE D2/3 RECEPTOR ANTAGONIST

**DOI:** 10.1093/schbul/sby017.707

**Published:** 2018-04-01

**Authors:** Kasper Jessen, Egill Rostrup, Rene C W Mandl, Mette Ø Nielsen, Nikolaj Bak, Birgitte Fagerlund, Birte Y Glenthøj, Bjørn H Ebdrup

**Affiliations:** 1 Center for Clinical Intervention and Neuropsychiatric Schizophrenia Research, Mental Health Centre Glostrup, University of Copenhagen; 2 Brain Center Rudolf Magnus, University Medical Center Utrecht

## Abstract

**Background:**

Schizophrenia has been associated with changes in both cortical thickness and surface area, but antipsychotic exposure, illness progression, and substance use may confound the observations. We investigated cortical thickness and surface area, as well as mean curvature, before and after antipsychotic monotherapy with a relatively selective dopamine D2/3 antagonist (amisulpride), in antipsychotic-naïve schizophrenia patients.

**Methods:**

Fifty-six patients and 59 matched healthy controls (HC) underwent T1-weighted 3T magnetic resonance imaging. Forty-one patients and 51 HCs were re-scanned. FreeSurfer-processed baseline values and symmetrized percentage changes (SPC) in cortical structures were analysed using univariate ANOVA. Clinical measures comprised psychopathology ratings, assessment of functioning, and tests of premorbid and current intelligence.

**Results:**

At baseline, groups did not differ in cortical thickness or surface area, while left curvature was higher in patients (p=0.015). In the complete sample, a higher curvature was associated with lower premorbid- (p=0.009) and current intelligence (p<0.001). Also, a lower surface area was associated with lower premorbid intelligence (p=0.014) and, in patients, a higher PANSS total (p=0.037). Lifetime substance use was associated with reduced cortical thickness (p=0.043). After six weeks, groups did not differ in overall change in cortical structures. Amisulpride dose (275.0 mg/day) did not correlate with cortical structures (p>0.43). A decrease in cortical thickness SPC was associated with less symptom improvement (p=0.002) and increase in surface area SPC was associated with improvement in GAF (p=0.047).

**Discussion:**

Our results indicate that schizophrenia may be associated with subtle aberrations in cortical structures and these changes appear clinically relevant. Mean curvature holds promise as a sensitive supplement to cortical thickness and surface area, to detect complex structural brain abnormalities.

